# The Difference in Clinical Features and Hepatic Injury Characteristics Between Children and Adults With Infectious Mononucleosis

**DOI:** 10.1002/jgh3.70382

**Published:** 2026-03-24

**Authors:** Ruiyun Sun, Xiaoqian Zhang, Junyan Qiao, Xin Nan, Chang Liu, Yan Yang, Danlei Mou

**Affiliations:** ^1^ Department of Infectious Diseases, Beijing Youan Hospital Capital Medical University Beijing China; ^2^ Department of Chinese Medicine, Beijing Children's Hospital, National Center for Children's Health Capital Medical University Beijing China; ^3^ Department of Chinese Medicine Haidian District Maternal and Child Health Care Hospital Beijing China

**Keywords:** adults, children, clinical manifestations, hepatic injury, infectious mononucleosis

## Abstract

**Aims:**

We aimed to analyze the characteristics of clinical features and hepatic injury characteristics of IM in different age groups.

**Methods:**

A retrospective study was conducted on IM patients hospitalized in Beijing Youan Hospital, Capital Medical University from July 2015 to July 2025. General data, clinical features, and laboratory information were collected.

**Results:**

Two hundred patients were enrolled, among them 49.5% were children, and 50.5% were adults. The results indicated that adults had longer fever duration (9 vs. 7 days) and hospitalization days (8 vs. 7 days), a higher proportion of splenomegaly (76.24% vs. 46.46%) and hepatic injury (99.01% vs. 67.68%) (all *p* < 0.05). Significant differences were observed between the two groups in the level of ALT (368 vs. 90 U/L) and AST (227 vs. 68 U/L) (all *p* < 0.05). No significant difference was observed between groups regarding gender, maximum temperature, sore throat, decreased appetite, tonsils covered with white membrane, hepatomegaly, hilar lymphadenopathy, leukocytes count, lymphocytes count, CRP, procalcitonin, albumin, EBV‐DNA, short lymph node diameter, CD4+ T cells, and CD8+ T cells (all *p* > 0.05).

**Conclusions:**

Both adults and children typically present with the classic triad of IM. However, adults are more prone to prolonged fever, more prominent lymphadenopathy, and an increased incidence of splenomegaly. Hepatic injury is a common complication of IM, occurring with higher incidence and greater severity in adults compared to children; adults more frequently present with concurrent cholestasis and have a predisposition to developing severe liver injury.

## 1 Introduction

Infectious mononucleosis (IM) is an acute systemic disease resulting from primary Epstein–Barr virus (EBV) infection, and may present with typical clinical manifestations, including the triad of fever, pharyngitis, and cervical lymphadenopathy, which may be accompanied by abnormal liver function, hepatosplenomegaly, eyelid edema, and rash [1]. In severe cases, however, IM can progress to acute liver failure (ALF) [2, 3]. Although EBV is not a hepatotropic virus, it remains an important and non‐negligible cause of hepatic injury. Research has shown that 40%–48% of children with IM present with hepatic injury, and the risk of such injury increases with age [4, 5]. Specifically, the incidence of hepatic injury in adolescent and adult patients with IM can be as high as 82.3%–97.4% [6, 7]. The characteristics of primary EBV infection are influenced by age, geographic region, race, and ethnicity. Previous studies have indicated that IM was a common acute infectious disease in children, less prevalent in adults. As a result, the majority of clinical and epidemiological studies on IM have focused predominantly on pediatric populations rather than adults. However, data on age‐specific differences in clinical manifestations and hepatic injury severity of IM remain limited, especially regarding high‐risk factors for hepatic damage. Additionally, existing studies on IM‐associated hepatic injury have primarily focused on elevated transaminase levels, without classifying the severity of such injury. In this study, we preliminarily analyzed the clinical characteristics and hepatic injury features of patients with IM across different age groups. Our findings may contribute to enhancing the understanding of IM in adults and hepatic injury as its complication.

## 2 Methods

### 2.1 Participants

This was a retrospective analysis. Inpatients hospitalized between July 1, 2015 and July 1, 2025 were enrolled by the medical record system of hospital. Patients were divided into a children group and an adult group according to age. This study was approved by the Ethics Committee (No.: LL‐2023‐84‐K). All procedures performed in this study involving human participants were in accordance with the principles laid down in the Declaration of Helsinki.

### 2.2 Inclusion Criteria

The inclusion criteria were as follows. Cases conforming to the Expert Consensus on the Diagnostic and Therapeutic Principles for Epstein–Barr Virus Infection‐Related Diseases in Children [8]. For the clinical diagnosis of IM, patients had to meet any three of the following clinical manifestations: fever, pharyngitis, cervical lymphadenopathy, hepatomegaly, splenomegaly, eyelid edema; patients also had to satisfy either of the following: a proportion of atypical lymphocytes ≥ 10% or, a peripheral blood lymphocyte proportion > 50% or an absolute lymphocyte count > 5.0 × 10^9^/L.

### 2.3 Exclusion Criteria

The exclusion criteria were: (1) incomplete patient data; and (2) patients had chronic diseases such as hematological diseases, tumors, or were newly diagnosed with liver diseases (including hepatitis A, hepatitis B, hepatitis C, cirrhosis, autoimmune liver diseases).

### 2.4 Measures

We collected data including age, gender, disease duration, clinical symptoms, signs, length of hospital stay, complications, and treatment regimens. Auxiliary examinations encompassed routine blood tests, C‐reactive protein (CRP), procalcitonin, liver function, atypical lymphocyte ratio, EBV antibody, serum EBV DNA load (EBV‐DNA), cervical lymph node B‐ultrasound, and abdominal B‐ultrasound. All patients underwent blood sampling on the day of admission; samples were sent to the laboratory department of our hospital.

The reference range for normal liver function in this study was as follows: alanine aminotransferase (ALT) and aspartate aminotransferase (AST) 0–40 U/L, serum total bilirubin (TBiL) 5–21 μmol/L, and γ‐glutamyl transferase (γ‐GT) 10–60 U/L. Hepatic injury is defined as abnormal levels of ALT and/or AST, and may be accompanied by derangements in associated laboratory parameters including bilirubin and coagulation function [9]. The severity assessment of hepatic injury mainly relies on ALT. Mild hepatic injury is generally defined as ALT levels satisfying 1 upper limit of normal (ULN; 1 ULN, 40 U/L) ≤ ALT < 5 ULN. Moderate hepatic injury is generally defined as 5 ULN ≤ ALT < 15 ULN. Severe hepatic injury is defined as INR ≥ 2.0, ALT ≥ 10 × ULN, TBiL ≥ 3.0 mg/dL, and absence of hepatic encephalopathy [10].

### 2.5 Statistical Analysis

Measurement data conforming to a normal distribution are reported as mean ± standard deviation and those not conforming to a normal distribution are presented as median (IQR). Categorical data are expressed as frequency (percentage). In the statistical analysis, between‐group comparisons were performed as follows: for measurement data with a normal distribution, the *t*‐test was used; for non‐normally distributed measurement data, the rank‐sum test was applied. For categorical data, comparisons between groups were conducted using the chi‐square test or Fisher's exact test, as appropriate.

## 3 Results

### 3.1 General Demographic Data and Clinical Characteristics

Of the 200 pertussis cases, 118 were males and 82 were females. Among them, 50.5% (101/200) were adults, 49.5% (99/200) were children. The median age of adults was 24 years (IQR: 21–29), which of children was 8 years (IQR: 4–16). As shown in Table 1, summarizes differences in the demographic and clinical characteristics by age groups. No significant difference was observed between groups with regard to gender (*χ* ^2^ = 0.029, *p* = 0.865), maximum temperature (*Z* = 0.338, *p* = 0.735), sore throat (*χ* ^2^ = 0.459, *p* = 0.498), decreased appetite (*χ* ^2^ = 0.956, *p* = 0.328), tonsils covered with a white membrane (*χ* ^2^ = 1.059, *p* = 0.589), hepatomegaly (*χ* ^2^ = 0.806, *p* = 0.369), hilar lymphadenopathy (*χ* ^2^ = 0.071, *p* = 0.79), or antiviral treatment with acyclovir or ganciclovir (*χ* ^2^ = 0.478, *p* = 0.489). However, significant differences were observed between the adult group and children group in fever duration (9 vs. 7 days, *Z* = −1.962, *p* = 0.049), proportion of enlarged tonsils (81.1% vs. 94.94%, *χ* ^2^ = 8.982, *p* = 0.03), palpebral edema (28.71% vs. 58.59%, *χ* ^2^ = 18.153, *p* < 0.001), rash (5.94% vs. 17.17%, *χ* ^2^ = 6.196, *p* = 0.013), hepatic injury (99.01% vs. 67.68%, *χ* ^2^ = 35.626, degrees of freedom = 1, *p* < 0.001), and days of hospitalization (8, IQR 6–11 vs. 7, IQR 6–8; Z = −2.047, *p* = 0.041). Abdominal B‐ultrasound findings showed that the incidence of splenomegaly was higher in the adult group than that in the children group (76.24% vs. 46.46%, *χ* ^2^ = 18.717, degrees of freedom = 1, *p* < 0.001), with a statistically significant difference (*p* < 0.05) (Table 1).

**TABLE 1:** Baseline data, clinical symptoms, and treatment status of the two groups of patients with IM.

Project	Adult group (*n* = 101)	Children group (*n* = 99)	*Z*/*χ* ^2^	*p*
Gender (male/female)	59/42	59/40	0.029	0.865
Male	59 (58.42)	59 (59.6)		
Female	42 (41.58)	40 (40.4)		
Age	24 (21, 29)	8 (4, 16)	—	—
*T*_max_	38.5 (38.0, 39.2)	38.6 (38.25, 39)	0.338	0.735
Fever duration	9 (7, 14)	7 (5, 11)	−1.962	0.049
Sore throat	90 (89.11)	91 (91.92)	0.459	0.498
Decreased appetite	85 (84.16)	78 (78.79)	0.956	0.328
Enlarged tonsils	82 (81.1)	94 (94.94)	8.982	0.03
I	16 (15.84)	19 (19.19)		
II	60 (59.41)	68 (68.69)		
III	6 (5.94)	7 (7.07)		
Covered with a white membrane	72 (71.29)	72 (72.73)	1.059	0.589
Palpebral edema	29 (28.71)	58 (58.59)	18.153	< 0.001
Rash	6 (5.94)	17 (17.17)	6.196	0.013
Hepatomegaly	5 (4.95)	8 (8.08)	0.806	0.369
Hilar lymphadenopathy	23 (22.77)	21 (21.21)	0.071	0.79
Splenomegaly	77 (76.24)	46 (46.46)	18.717	< 0.001
Hepatic injury	100 (99.01)	67 (67.68)	35.626	< 0.001
Antiviral treatment with Acyclovir or ganciclovi	35 (38.89)	20 (33.33)	0.478	0.489
Hospitalization days	8 (6, 11)	7 (6, 8)	−2.047	0.041

*Note:* Values are median (interquartile range) or *n* (%). *p* values calculated using the rank‐sum test or *χ* ^2^ test, as applicable.

Abbreviations: IM, infectious mononucleosis; *T*_max_, maximum temperature.

### 3.2 Laboratory Findings and B‐Ultrasound Examination Results in the Two Groups

In laboratory testing, no significant difference was observed between the adult group and children group regarding leukocytes count (11.82 × 10^9^/L vs. 11.5 × 10^9^/L, *Z* = 0.567, *p* = 0.571), lymphocytes count (7.34 × 10^9^/L vs. 7.63 × 10^9^/L, *Z* = 0.726, *p* = 0.468), proportion of lymphocyte (69.7% vs. 68.4%, *Z* = 0.188, *p* = 0.851), CRP (5 vs. 5 mg/L, *Z* = −1.236, *p* = 0.216), procalcitonin (0.11 vs. 0.12 ng/L, *Z* = −0.251, *p* = 0.802), albumin (39.7 vs. 40 g/L, *Z* = 1.35, *p* = 0.177), EBV‐DNA (5190 vs. 3900 copies/mL, *Z* = −0.959, *p* = 0.337), short diameter of lymph nodes (9 vs. 9 mm, *Z* = −0.709, *p* = 0.479), CD4+ T cells (*Z* = 0.063, *p* = 0.950), and CD8+ T cells (*t* = 0.076, *p* = 0.940). However, significant differences were observed between the two groups in the proportion of atypical lymphocytes (23% vs. 14%, *Z* = −2.806, *p* = 0.005), ALT (368 vs. 90 U/L, *Z* = −6.43, *p* < 0.001), AST (227 vs. 68 U/L *Z* = −6.219, *p* < 0.001), TBiL (15.6 vs. 8.7 μmol/L, *Z* = −6.688, *p* < 0.001), γ‐GT (144.5 vs. 98 U/L, *Z* = −2.546, *p* < 0.011), the seropositivity rates for VCA‐IgM (51% vs. 76%, *Z* = 9.875, *p* = 0.002), and long diameter of lymph nodes (24 vs. 22 mm, *Z* = −2.182, *p* = 0.029) (Table 2). Among them, 99.01% (100/101) adult infectious mononucleosis patients were diagnosed hepatic injury, and 67.68% (67/99) children IM patients were diagnosed with hepatic injury. There were 42 cases and 70 cases in adult group with elevated TBiL and γ‐GT levels, and there were 8 cases and 27 cases in children group with elevated levels of TBiL and γ‐GT, respectively. The differences between the two groups were statistically significant (*p* < 0.05). EBV‐DNA positivity was detected in 181 cases (90.5%), with values ranging from 5.01 × 10^2^ to 9.43 × 10^5^ copies/mL (normal value: < 5.0 × 10^2^ copies/mL). There were no significant differences in EBV‐DNA levels between the two groups (*p* > 0.05). Cervical lymph nodes were 24 × 9 mm in the adult group, and were 22 × 9 mm in the children group (*p* < 0.05) (Table 2).

**TABLE 2:** Laboratory and B‐ultrasound results in children and adult patients with IM.

Indicator	Adult group (*n* = 101)	Children group (*n* = 99)	*t*/*Z*/*χ* ^2^	*p*
Leukocyte count (×10^9^/L)	11.82 (9.10, 14.14)	11.5 (9.51, 16.00)	0.567	0.571
Lymphocyte count (×10^9^/L)	7.34 (5.92, 9.42)	7.63 (6, 9.99)	0.726	0.468
Proportion of lymphocyte (%)	69.7 (62.8, 76.3)	68.4 (62.9, 76)	0.188	0.851
Proportion of atypical lymphocytes (%)	23 (12, 32)	14 (10, 22.5)	−2.806	0.005
CRP (mg/L)	5 (3, 10)	5 (3, 6.15)	−1.236	0.216
PCT^a^ (ng/L)	0.11 (0.05, 0.2)	0.12 (0.05, 0.2)	−0.251	0.802
ALT (U/L)	368 (213, 520.6)	90 (33.5, 301.5)	−6.43	< 0.001
AST (U/L)	227 (128, 343)	68 (29.5, 199)	−6.219	< 0.001
TBiL (μmol/L)	15.6 (9.9, 35.4)	8.7 (6.05, 12.6)	−6.688	< 0.001
ALB (g/L)	39.7 (37.2, 41.6)	40 (38.3, 41.5)	1.35	0.177
γ‐GT^b^ (U/L)	144.5 (77.75, 216)	98 (36, 169)	−2.546	0.011
EBV‐DNA (copies/mL)	5190 (1390, 12 600)	3900 (1050, 10 450)	−0.959	0.337
VCA‐IgM (+) [case (%)]	51 (73.91)	76 (92.68)	9.875	0.002
Cervical lymphadenopathy (mm)	101 (100)	99 (100)	—	—
Long diameter of lymph nodes	24 (19, 28)	22 (18, 26)	−2.182	0.029
Short diameter of lymph nodes	9 (8, 11)	9 (7, 10.5)	−0.709	0.479
CD4+ T cells^c^	571 (464, 629.5)	519 (488, 601)	0.063	0.950
CD8+ T cells^c^ (mean ± SD)	3936.13 ± 2327.41	4014.78 ± 3286.04	0.076	0.940

*Note:* Values are median (interquartile range), or *n* %. *p* values calculated using the *t*‐test, rank‐sum test, or *χ* ^2^ test, as applicable.

Abbreviations: γ‐GT, γ‐glutamyl transferase; ALB, albumin; ALT, alanine aminotransferase; AST, aspartate aminotransferase; CRP, C‐reactive protein; DBil, direct bilirubin; EBV, Epstein–Barr virus; IM, infectious mononucleosis; PCT, procalcitonin; SD, standard deviation; TBiL, total bilirubin; VCA‐IgM, viral capsid antigen immunoglobulin M.

^a^ In terms of specific test data: 38 patients in the children group and 84 in the adult group underwent PCT testing.

^b^ In terms of specific test data: 45 patients in the children group and 80 in the adult group were tested for γ‐GT.

^c^ In terms of specific test data: nine cases in the children group and 23 in the adult group were tested for CD4+ T cells.

The main manifestations of hepatic injury are elevated ALT and/or AST, with some patients also having elevated bilirubin. Children with IM predominantly presented with mild hepatic injury (ALT/AST < 200 U/L), while 55.26% of adolescents and 60% of adults had moderate hepatic injury (200 U/L ≤ ALT/AST < 600 U/L). In contrast, adult patients demonstrated a predisposition to severe hepatic injury, with 39.25% of this group presenting with elevated bilirubin levels (Table 3).

**TABLE 3:** Degree of hepatic injury and population distribution in 167 patients with IM and liver function impairment.

Classification of hepatic injury	Indicator	Adults (*n* = 100)	Children (*n* = 67)		*χ* ^2^	*p*
			≤ 12 years old (*n* = 29)	Adolescents^a^ (*n* = 38)		
ALT or AST < 5 × ULN	ALT	23 (23.0)	19 (65.52)	16 (42.10%)	21.41	< 0.001
	AST	45 (45.00)	21 (72.41)	18 (47.37)	8.42	0.02
5 × ULN ≤ (ALT or AST) < 15 × ULN	ALT	60 (60.00)	8 (27.59)	21 (55.26)	10.35	0.005
	AST	50 (50.00)	7 (24.14)	20 (52.63)	9.14	0.01
ALT or AST ≥ 15 × ULN	ALT	17 (17.00)	2 (6.90)	3 (9.68)	2.87	0.24
	AST	5 (5.00)	1 (3.45)	0 (0)	1.52	0.47
	TBiL	42 (39.25)	2 (6.90)	6 (19.35)	13.42	0.001
	DBil	49 (32.26)	3 (10.34)	10 (32.26)	12.67	0.002
	γ‐GT	70 (65.42)	10 (34.48)	17 (54.84)	9.13	0.01

*Note:* Values are *n* (%). ULN is 40 U/L.

^a^ Children aged 12–18 years were defined as adolescents.

## 4 Discussion

EBV, a member of the herpesvirus family, is transmitted via saliva, with > 95% of the global population infected [11, 12]. The incidence of IM across different age groups varies among countries owing to differences in economic and hygiene conditions. In developing countries, the peak age of primary EBV infection occurs during school age, which is earlier than in developed countries such as the United States and European countries. In China, children aged 4–6 years have the highest incidence of IM as well as the highest hospitalization rate among all age groups, with an average hospital stay of 8 days, which is consistent with our study findings [13, 14].

As economic and educational development progresses, children are acquiring EBV infection at older ages in developing countries [15]. Studies showed an increased prevalence of IM among adolescents and young adults, particularly those aged 15–24 years [16]. Our study enrolled 90 cases (45%) in the 15–24 years age group, comprising high school and college students. Previous studies conducted in the United Kingdom and the United States showed that the incidence of primary EBV infection among college students was 46%, while the incidence of IM rose from 24.5% to 77% [17, 18]. Further studies confirmed that the only significant risk factor for acquisition of EBV infection was deep kissing [18]. Several prospective studies have documented that the incidence of primary EBV infection during the four undergraduate years was 14.4–15.2 cases per 100 person‐years [17, 18], and this indicated college students account for a high proportion in IM patients. Consistent with previous research, the epidemiology of EBV infection also shows a trend toward an older age at onset. Viral capsid antigen immunoglobulin M is an indicator of acute EBV infection, with studies reporting the highest positive rate among patients aged 11–20 years [19, 20]. The increasing incidence of IM in adults and rising rate of hospitalization for IM warrant greater attention [21].

Hepatic injury is a common complication of IM, with some patients presenting at their initial visit [22, 23]. ALT and AST are sensitive indicators of hepatic injury, and hepatic injury may lead to elevated bilirubin levels. The γ‐GT plays an auxiliary role in biliary excretion, with elevated bilirubin and γ‐GT levels indicating cholestasis. Previous studies showed that hepatic injury in children with IM is mainly characterized by mild elevations in ALT and/or AST, with no abnormalities in bilirubin or bile acids [24]; however, adults may develop hepatic injury characterized by cholestatic hepatitis and hyperbilirubinemia [25]. Hepatic injury in IM is not caused by viral infection itself but may result from immune injury due to lymphocyte infiltration or overactivated immune responses following EBV infection [26, 27]. Studies found that hepatic injury in children with IM was transient and mild, with severe elevations in transaminases (5–10 times the ULN) being rare [28]. In our study, 167 cases have elevated transaminases, with 111 (66.46%) showing ALT levels exceeding five times the ULN and 83 patients (49.70%) with AST levels exceeding five times the ULN. This may be attributed to the age‐related nature of hepatic injury; we included a large proportion of adults, resulting in more moderate hepatic injury.

The incidence of hepatomegaly and splenomegaly in children with IM was 45%–70% and 35%–50%, respectively; in adolescents and adults with IM, these figures are approximately 50% and 10% [11, 16]. In our study, 76.24% of adults had splenomegaly, which was higher than the rate reported in previous research, suggesting that IM was not a trivial illness especially for adults. EBV is a common cause of cervical lymphadenopathy; we also found that EBV infection can cause hilar lymphadenopathy. The hilar region is an important passage for hepatic blood vessels, bile ducts, and lymphatic vessels entering and exiting the liver. Both hepatomegaly and hilar lymphadenopathy can be caused by EBV infection, and these may exhibit different degrees of liver changes associated with the severity of IM. Spontaneous splenic rupture is a fatal complication of IM in adolescents, with an incidence of 0.1%–0.5%. ALF is a cause of death that requires vigilance in adults with IM [4], and EBV‐associated ALF accounts for 0.2% of all ALF cases [29, 30]. The mortality rate of liver failure ranges from 60% to 80%, depending on the cause and medical conditions [3]. So, clinicians should pay close attention to the detection and evaluation of liver function in adolescent and adult patients with IM.

Several studies elaborated on the risk factors for IM complicated with hepatic injury from different perspectives. Studies found that a peripheral blood lymphocyte count ≥ 5 × 10^9^/L was an independent risk factor for hepatic injury in adults with IM [6]. Additionally, other studies indicated that age, gender, the proportion of atypical lymphocytes, duration of fever (> 7 days), and serum EBV‐DNA levels were associated with the occurrence of hepatic injury [3, 6, 24], emphasizing the importance of early stratified management based on EBV‐DNA load to prevent complications [31]. Some scholars have studied the relationship between immune function and hepatic injury, revealing that immunoglobulins G and A, CD4^+^ T lymphocytes, and CD8^+^ T lymphocytes were associated with the development of hepatic injury [24]. Among numerous studies related to hepatic injury, age has a relatively high correlation with hepatic injury. In our study, the incidence and severity of hepatic injury as well as the length of hospital stay in the adult group were higher than those in the children group, suggesting that hepatic injury may increase the disease burden of IM.

Administering hepatoprotective and enzyme‐lowering therapy to patients with hepatic injury can improve their condition. According to some studies, hepatitis due to primary EBV infection was mostly benign, and recovery of liver function in patients with IM can occur within 5–12 weeks [32, 33]. Whether combined antiviral therapy is beneficial remains unclear; however, routine use of antivirals and corticosteroids was not recommended [34, 35]. Some scholars believe that abnormal liver function does not affect decision‐making regarding antiviral therapy [36]. Although some studies suggest that antiviral therapy should be administered in the early stage of severe IM, the elaboration definition of severe IM lacks [37]. Moreover, antiviral drugs such as acyclovir and ganciclovir may increase the burden on the liver; therefore, antiviral drugs should be used with caution in patients with IM who have hepatic injury.

### 4.1 Limitations

This study has certain limitations. As this is a single‐center study from a specialized liver/infectious disease hospital, the incidence of hepatic injury may be overestimated. Going forward, multi‐center, all‐age clinical studies on IM should be conducted to comprehensively assess clinical characteristics of the broader IM population. Nonetheless, our findings may contribute to the improvement of knowledge of adults with IM and hepatic injury as a complication of IM.

## 5 Conclusion

Both adults and children commonly present with the classic triad of fever, pharyngitis, and cervical lymphadenopathy, yet distinct age‐related differences exist in clinical manifestations. Adults tend to experience prolonged fever, longer hospitalization durations, more prominent lymphadenopathy, and a higher incidence of splenomegaly. In contrast, children exhibit a higher proportion of eyelid edema, rash, and tonsillar hypertrophy. Notably, hepatic injury is a common complication of IM, with adults showing a higher incidence and greater severity than children. Specifically, among hospitalized IM patients in certain regions of China, adults constitute a substantial proportion. Hepatic injury, a prevalent complication of IM, occurs with higher incidence and greater severity in hospitalized adults than in children. The primary manifestation of such hepatic injury is elevated alanine transaminase (ALT) and/or aspartate transaminase (AST): children predominantly presented with mild hepatic injury, whereas adults were characterized by moderate hepatic injury and have a predisposition to developing severe hepatic injury.

## Funding

This work was supported by the National Key Research and Development Program (Approval No. 2022YFC2304404); Special Projects of World Federation of Chinese Medicine Societies (Approval No. WFCMS2024052); and the Scientific Research Project of hospital (BJYAYY‐YN2022‐11).

## Ethics Statement

This study was approved by the Ethics Committee of Beijing Youan Hospital, Capital Medical University (Ethics No.: LL‐2023‐84‐K), in accordance with the Declaration of Helsinki. The study cohort comprised individuals under the age of 16 years. Prior to study initiation, written informed consent was obtained from all participants' parents or legal guardians in accordance with ethical guidelines.

## Consent

The authors have nothing to report.

## Conflicts of Interest

The authors declare no conflicts of interest.

## Data Availability

The data that support the findings of this study are available on request from the corresponding author. The data are not publicly available due to privacy or ethical restrictions.
